# Supplementary figures and images for: A robust transcriptional program in newts undergoing multiple events of lens regeneration throughout their lifespan
Source: eLife. 2015 Nov 2;4:e09594. doi: 10.7554/eLife.09594 (PMC4739772; doi:10.7554/eLife.09594)

## Color Key

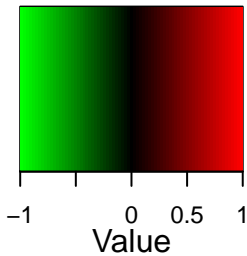

sample correlation matrix  
diffExpr.P1e-3\_C2.matrix.log2 (jackknifing)

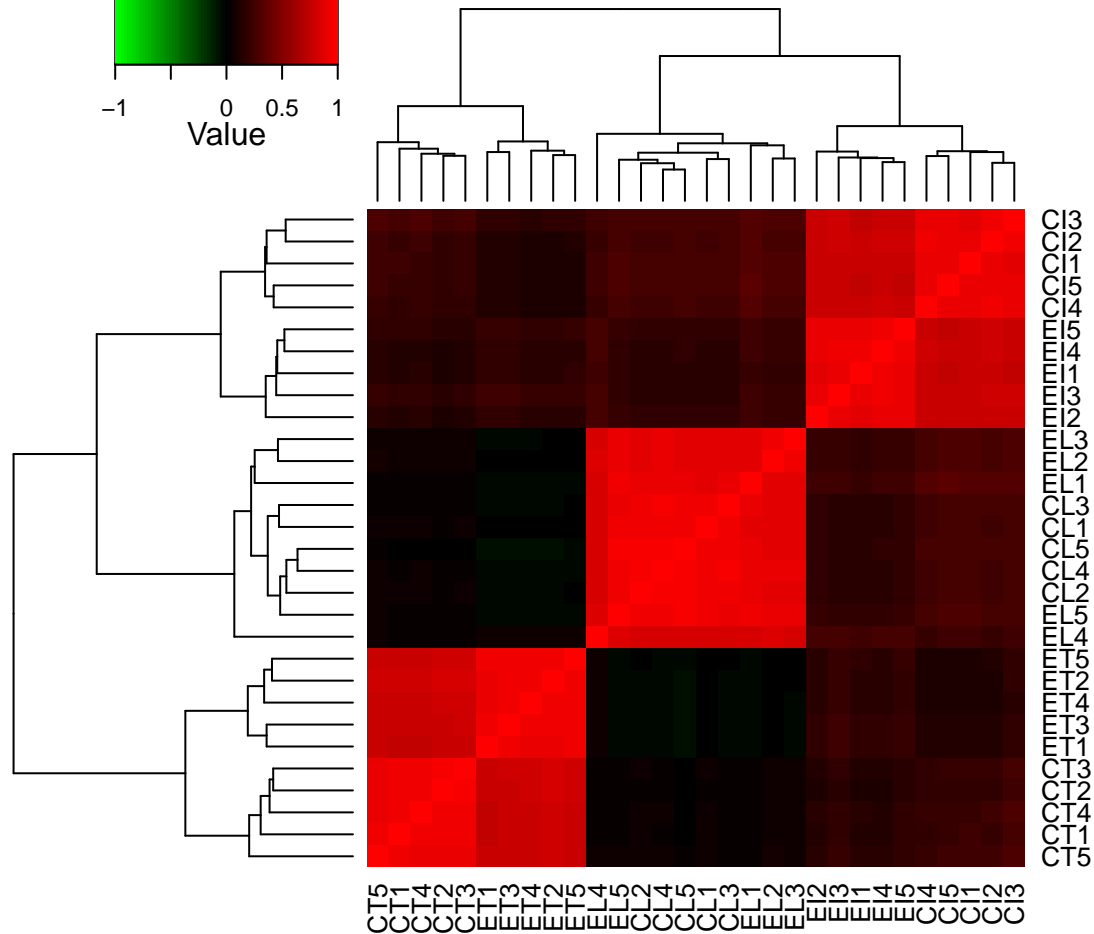

Supplement: Supplementary file 7. — DOI: http://dx.doi.org/10.7554/eLife.09594.016 [file elife-09594-supp7.zip › Supplementary_file_7.pdf]

# Color Key

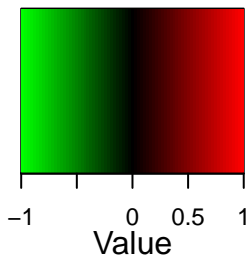

sample correlation matrix  
diffExpr.P1e-3\_C2.matrix.log2 (20% sampling)

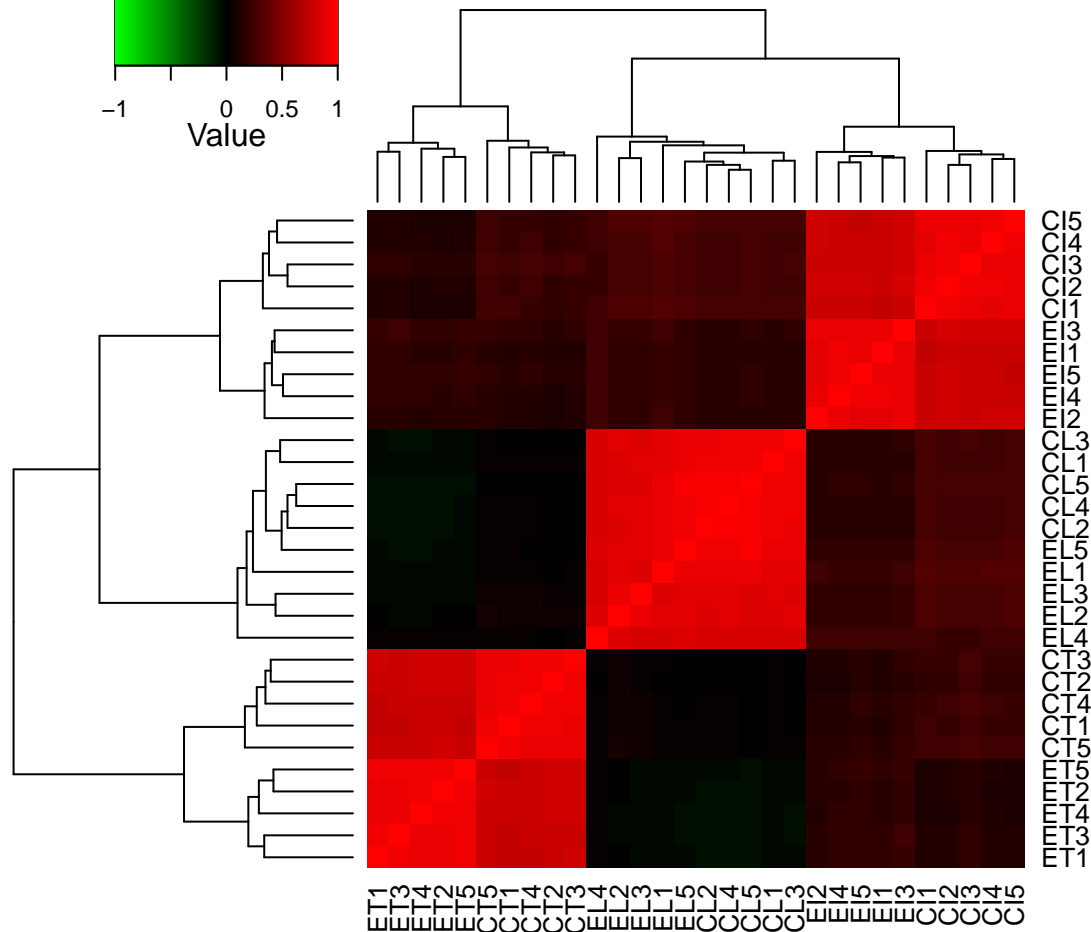

Supplement: Supplementary file 8. — DOI: http://dx.doi.org/10.7554/eLife.09594.017 [file elife-09594-supp8.zip › Supplementary_file_8.pdf]
